# Pixel-level multimodal fusion deep networks for predicting subcellular organelle localization from label-free live-cell imaging

**DOI:** 10.3389/fgene.2022.1002327

**Published:** 2022-10-26

**Authors:** Zhihao Wei, Xi Liu, Ruiqing Yan, Guocheng Sun, Weiyong Yu, Qiang Liu, Qianjin Guo

**Affiliations:** ^1^ Academy of Artificial Intelligence, Beijing Institute of Petrochemical Technology, Beijing, China; ^2^ School of Mechanical Engineering & Hydrogen Energy Research Centre, Beijing Institute of Petrochemical Technology, Beijing, China

**Keywords:** label-free live cell imaging, protein subcellular localization, non-linear optical microscopy, Transformer–Unet network, deep learning

## Abstract

Complex intracellular organizations are commonly represented by dividing the metabolic process of cells into different organelles. Therefore, identifying sub-cellular organelle architecture is significant for understanding intracellular structural properties, specific functions, and biological processes in cells. However, the discrimination of these structures in the natural organizational environment and their functional consequences are not clear. In this article, we propose a new pixel-level multimodal fusion (PLMF) deep network which can be used to predict the location of cellular organelle using label-free cell optical microscopy images followed by deep-learning-based automated image denoising. It provides valuable insights that can be of tremendous help in improving the specificity of label-free cell optical microscopy by using the Transformer–Unet network to predict the ground truth imaging which corresponds to different sub-cellular organelle architectures. The new prediction method proposed in this article combines the advantages of a transformer’s global prediction and CNN’s local detail analytic ability of background features for label-free cell optical microscopy images, so as to improve the prediction accuracy. Our experimental results showed that the PLMF network can achieve over 0.91 Pearson’s correlation coefficient (PCC) correlation between estimated and true fractions on lung cancer cell-imaging datasets. In addition, we applied the PLMF network method on the cell images for label-free prediction of several different subcellular components simultaneously, rather than using several fluorescent labels. These results open up a new way for the time-resolved study of subcellular components in different cells, especially for cancer cells.

## 1 Introduction

For cell biology, cell function determined by its variety of organelles and subcellular structures is the central conjecture. Therefore, determining the subcellular organization is very important for elucidating the cell state, as well as the response to environmental perturbations or mutations (Koenig et al., 2001; Szabo et al., 2014; Mottis et al., 2019; Yuan et al., 2019; Parlakgül et al., 2022). However, the resolution of the subcellular structure in a natural tissue environment and its functional consequences are still challenging, which are largely decided by the large amount of different molecules, complexes, and organelles that constitute living cells and influence their functions (Chou and Shen, 2007; Hung and Link, 2011; Xu et al., 2013; Guo et al., 2016). Accordingly, the capability of imaging, extracting, and exploring cells and their subcellular compartments is very essential in various research fields such as cell physiology and pathology and is closely related to a variety of diseases.

Based on the aforementioned reasons, various imaging tools of cell biology have been developed to overcome the limitations of the human eye and enable us to observe the structural and molecular adaptation of individual cells in their microenvironment (Chou and Shen, 2007; Hung and Link, 2011; Xu et al., 2013; Szabo et al., 2014; Guo et al., 2016; Ounkomol et al., 2018; Vicar et al., 2019; Zhang et al., 2019). These imaging methods mainly include mass spectrometry, emerging microscopy technologies such as electron microscopy, atomic-force microscopy, and different types of optical-imaging technologies such as fluorescence-imaging technology, confocal-microscopy imaging, phase-contrast imaging, Raman-imaging technology, and super-resolution fluorescence microscopy, which are extensively applied in unveiling cellular states and offer an important way to study different angles of cell information at high spatial and temporal resolutions (Chou and Cai, 2004; Chou and Shen, 2010; Armenteros et al., 2017; Buggenthin et al., 2017; Hasan et al., 2017; Wei et al., 2018; Falk et al., 2019; Jing et al., 2020; Wang et al., 2022a; Thi Le et al., 2022).

The optical-based method for single-cell imaging is one of the most effective approaches to predict protein subcellular localization, which has certain properties such as high-detection sensitivity, high quality, and low cost, and tremendously boosts the proceedings of non-destructive cell research (Armenteros et al., 2017; Buggenthin et al., 2017; Hasan et al., 2017; Jing et al., 2020; Thi Le et al., 2022). Especially in the last few years, great amounts of label-free optical-imaging instruments such as bright field, phase, differential interference contrast (DIC), and stimulated Raman scattering (SRS) microscopy were developed and utilized for cell survey (Zhang et al., 2012; Armenteros et al., 2017; Buggenthin et al., 2017; Hasan et al., 2017; Jing et al., 2020; Thi Le et al., 2022). Compared with pathological images that need to be stained and fluorescent images requiring labeling, label-free optical imaging overcomes the unfavorable influence of staining reagents on cytoactive and cell-signal transduction, and can be used for long-time detection in tissues and living cells (Zhang et al., 2012; Armenteros et al., 2017; Hasan et al., 2017; Thi Le et al., 2022). On the other hand, it is difficult to analyze and extract effective features from the images collected by these label-free optical methods due to the rich information contained and spectral overlap. Therefore, there are increasing demands to develop advanced optical-imaging analysis methods for handling the specificity and clear separation of the structures of interest contained in the label-free cell images (Jiang et al., 2017; Kobayashi et al., 2017; Wei et al., 2019).

Although different types of predictors have been developed for specific subcellular localizations, the systematic predicting approaches are still missing for revealing valuable biological patterns from pixel-level values with high sensitivity and high accuracy. Capturing the non-linear, subtle, and inhomogeneous features of optical-based label-free cell images requires a high understanding of important visual variations, which is easier to achieve through deep learning (Kobayashi et al., 2017; Wei et al., 2019; Siu et al., 2020; Li et al., 2022). In comparison with conventional intelligence method, deep learning is able to perform a series of target recognitions, feature extraction, and analysis automatically, which makes it possible to automatically discover image–target features and explore feature levels and interaction (Chen et al., 2016; Siu et al., 2020; Ullah et al., 2021; Li et al., 2022). The learning-enhanced cell optical image-analysis model is capable of acquiring the texture details from low-level source images and achieve higher resolution improvement for the label-free cell optical-imaging techniques (Chen et al., 2016; Lee et al., 2020; Ullah et al., 2021; Ullah et al., 2022). The deep-learning pipeline of cell optical microscopy imaging can extract complex data representation in a hierarchical way, which is helpful to find hidden cell structures from the microscope images, such as the size of a single cell, the number of cells in a given area, the thickness of the cell wall, the spatial distribution between cells, and subcellular components and their densities (Boslaugh and Watters, 2008; Donovan-Maiye et al., 2018; Falk et al., 2019; Manifold et al., 2019; Rezatofighi et al., 2019; Yao et al., 2019; Zhang et al., 2019; Lee et al., 2020; Voronin et al., 2020; Zhang et al., 2020; Chen et al., 2021a; Gomariz et al., 2021; Manifold et al., 2021; Wang et al., 2022b; Islam et al., 2022; Kim et al., 2022; Melanthota et al., 2022; Rahman et al., 2022; Ullah et al., 2022; Witmer and Bhanu, 2022).

To address these technical limitations and challenges for deeply exploring the cellular structure and morphological information, we developed a pixel-level multimodal fusion (PLMF) deep network to predict immunofuorescent-like images using stimulated Raman scattering microscopy data. In our work, we find that the pixel-level multimodal fusion method which incorporates all the merit features, both high-resolution local detailed spatial information from CNN features and the global context information from transformers, presents a better way to predict the location of cellular organelle using label-free cell optical images compared with previous CNN-based self-attention methods. Moreover, it is demonstrated that subcellular structures could be more precisely reconstructed with the combination of transformer and Unet than both methods working individually. The model also has strong generalization ability and can be extended to be utilized by the new cell-imaging investigation.

## 2 Materials and methods

### 2.1 Experiment of the simultaneous stimulated Raman scattering and fluorescence microscopy

**FIGURE 1:**
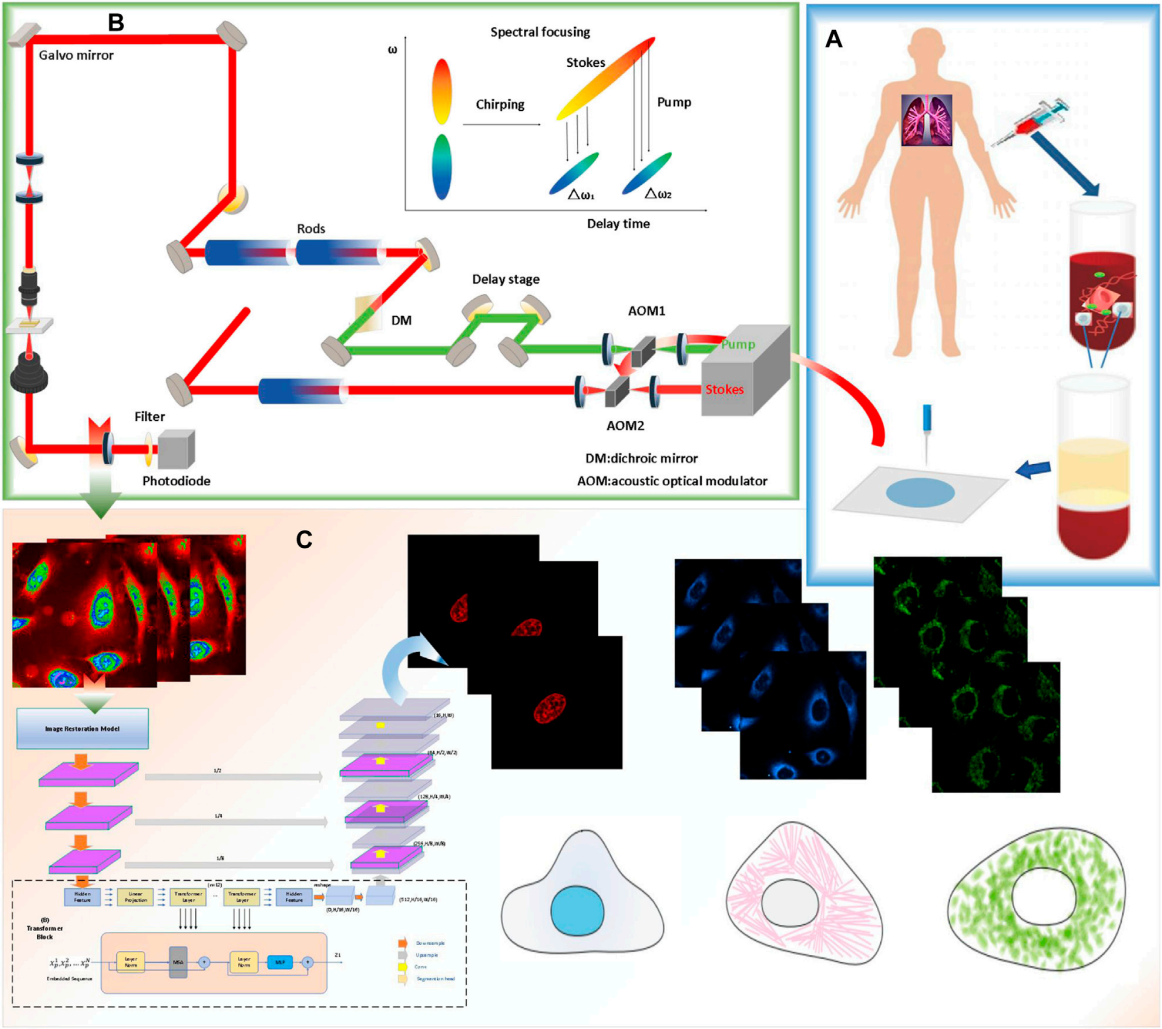
Workflow of the single-cell analysis by stimulated Raman scattering (SRS) imaging and deep-learning model prediction process. **(A)** cell sample is prepared. **(B)** The SRS signal and fluorescence signal of different lung cancer cell samples were collected simultaneously using the multimodel microscopy. **(C)** Protein subcellular localization based on Pixel Level Multimodal Fusion Deep Networks.

The complete experiment and process of predicting the protein’s subcellular localization based on a deep-learning network is shown in Figure 1. The deep-learning-based computer-aided method for detecting proteins’ subcellular localization using the stimulated Raman scattering (SRS) microscopic image framework consists of the following stages: the cell sample is first prepared. Later, the SRS signal and fluorescence signal of different lung cancer cell samples are collected simultaneously using stimulated Raman scattering microscopy. Finally, the protein subcellular localization of the lung cancer cell is performed using different machine-learning techniques.

Specifically, the lung cancer cells (A549, from ATCC) were first cultured in an ATCC F-12K medium. Then, the cells were fixed using 2% paraformaldehyde after being dyed. For the prepared live cells, after installing the living cell samples, the prepared cells were imaged with stimulated Raman scattering (SRS) microscopy. After that, fluorescence images of nuclei, mitochondria, and the endoplasmic reticulum were detected with fluorescent dyes of different colors. After denoising and enhancing the collected images, the processed cell-sampling image set is divided into two subsets, one of which is used for training, and the deep-learning algorithm based on different algorithms is used to train the model. Another subset is used as a test set and to validate the model.

### 2.2 Pixel level multimodal fusion deep network experiment

**FIGURE 2:**
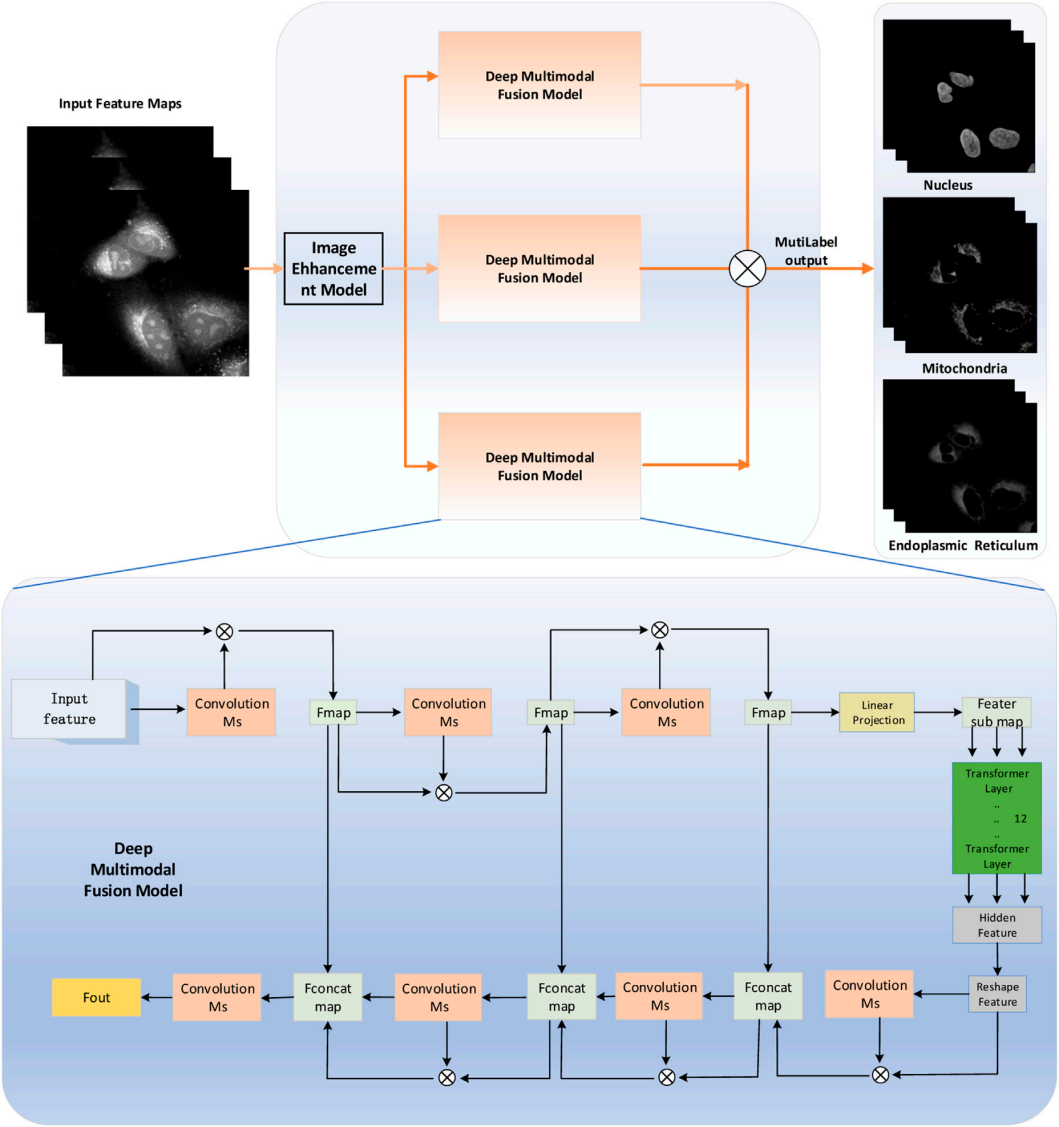
Pixel-level multimodal fusion deep networks for the protein subcellular localization from label-free live-cell imaging.

The bottleneck in predicting the protein subcellular locations of SRS cell imaging lies in modeling complicated relationships concealed beneath the original cell-imaging data owing to the spectral overlap information from different protein molecules. Concerned with the aforementioned issue, a pixel-level multimodal fusion (PLMF) deep network for the protein subcellular localization from label-free live cell-imaging is proposed to overcome the crowded and highly convoluted information as shown in Figure 2. The main processes are as follows:

Step 1 According to the lung cancer cell-imaging experiment, the lung cancer cell SRS-imaging data set was established and stored.

Step 2 Lung cancer cell SRS raw-data sequences for deep-learning-enabled image denoising and restoration were preprocessed.

Step 3 An integrated pixel-level multimodal fusion (PLMF) deep network framework was built.

Step 3.1 An independent transformer and CNN fusion models corresponding to different protein subcellular sites and fluorescence-imaging labels were constructed.

Step 3.2 Pixel-level multimodal fusion models were trained. The protein subcellular location prediction performance was evaluated according to the quantified metrics.

Step 4 The different cell data sets are applied to optimize the model parameters and find the optimal model combination.

Step 5 The protein subcellular sites are located by using new cell data.

### 2.3 Neural network architecture and implementation

**FIGURE 3:**
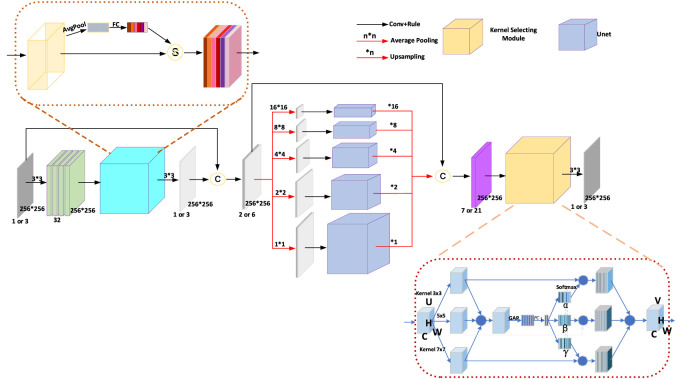
The architecture of the Pyramid Real Image Denoising Network (PRIDNet). The number of channels of the feature maps is shown below them; the symbol **C** indicates concatenation.

The overview diagram of the Transformer and Unet fusion model-based label-free organelle-prediction method from the optical microscopy images is shown in Figure 2. The performance of subcellular prediction depends largely on the feature extracted from the original cell images; the original input cell image is first fed into a multiscale filtering fusion-based convolutional neural network (CNN), which is used to remove noises from the raw cell images. Then, the fused image is fed into a Transformer and Unet fusion model-based network to obtain its corresponding cell fluorescence images for different subcellular organelles (seen in Figure 3; the nuclei, mitochondria, endoplasmic reticuli, etc.).

#### 2.3.1 Pyramid real-image denoising (PRID) network for image denoising and restoration

In non-linear optical-imaging technology, several aspects need to be overcome. On one hand, the light beam will be affected by absorption and scattering effects during the propagation of the sample tissue. On the other hand, in order to reduce the influence of the photo on the sample damage, which leads to the application of power limitation, it is necessary to comprehensively consider the factors such as sampling depth, laser power, and detection scheme, which often results in these obtained images having low signal-to-noise ratio. In the synthesis of the factor, all these challenges that include inadequate resolution, background noise, and scanning artifacts for the non-linear optical-imaging techniques often result in being susceptible to adverse effects and hinder their widespread application in cell optical imaging. Although different denoising methods have been developed to improve image quality in last few years, for conventional methods, when processing non-linear optical images, it is often difficult to clearly distinguish the relevant biological features, which is caused by the inability to recover its internal quantitative information. In addition, general denoising algorithms usually need *a priori* knowledge of interference noise or multiple images with the same characteristics to achieve an average, which usually leads to adverse consequences, such as the reduction of the effective spatial resolution of the image (Wang et al., 2004; Esakkirajan et al., 2019; Manifold et al., 2019).

Recently, deep-learning-based denoising tools which perform well in image-denoising work with induced Gaussian noise or inherent compression corruption as well as in blind denoising tests have shown great advantages and prospects (Hsieh et al., 2013; Zhang et al., 2018; Zhao et al., 2019). However, the most common CNN denoising model that is based on full-connection architecture often encounters some shortcoming to be solved, such as being unable to effectively remove the relative broadband noise, requiring a lot of training time and a large number of training samples to be effective (Zhang et al., 2018). In this work, several new CNN models, namely, the fast and flexible denoising convolutional neural network (FFDNet) and Pyramid Real Image Denoising Network (PRIDNet), for flexible, effective, and fast discriminative denoising, the PRIDNet is specifically presented in detail for blind denoising of cell images through three sequential stages (Al-Kofahi et al., 2018; Chen et al., 2021b; Fang et al., 2022).

As shown in Figure 3, the channel attention mechanism is first utilized in the noise-estimation stage for extracting the relative importance of feature channels hidden in the noisy image. For the input feature maps $U\in R^{H\times W\times C}$ , the key process is acquired the channel weight $\mu =\left[\mu_{1},\mu_{2},\ldots ,\mu_{c}\right]\epsilon R^{1\times 1\times C}$ for generating recalibrated features, which can be formulated as:

$$\mu =Sigmoid(FC_{2}\left(ReLU\left(FC1\left(GAP\left(U\right)\right)\right)\right)),$$ (1)

$$U^{\prime }=U\circ \mu ,$$ (2)

where $U^{\prime }\in R^{H\times W\times C}$ is the final output of the channel-attention module, ∘ refers to channel-wise multiplication between $U_{i}\in R^{H\times W}$ and scalar calibration weight $\mu_{i},\;i=1,2,\ldots ,C$ .

At the multi-scale denoising stage, five parallel-level pyramid pooling is applied to denoise multi-scale features, in which each branch pays attention to one-scale features, and each pooled feature is followed by U-Net that is composed of deep encoding–decoding and skip connections. The multi-level denoised features are finally upsampled by bilinear interpolation to the same size and then concatenated together. Benefitting from it, we can extract global information and retain local details simultaneously, thereby making preparations for the following comprehensive denoising.

At the last stage in Figure 3, multi-scale features are adaptively fused by selecting size-different kernel-selecting operation. For the input feature maps $V\in R^{H\times W\times C}$ , three feature branches $V^{\prime }\in R^{H\times W\times C}$ , $V^{\prime \prime }\in R^{H\times W\times C}$ ,and $V^{\prime \prime \prime }\in R^{H\times W\times C}$ can be acquired by using parallel convolutions on V with different kernel size 2 (k+1), k = 1,2,3. Then, all branches are summed by element-wise operation:

$$\bar{V}=V^{\prime }+V^{\prime \prime }+V^{\prime \prime \prime },$$ (3)

$\bar{V}$ is squeezed by passing through a global average pooling and then expanded by using two fully connected layers. The soft attention vector α, β , and γ for $U^{\prime }$ , $U^{\prime \prime }$ ,and $U^{\prime \prime \prime }$ can be computed as follows:

$$\alpha_{c}=\frac{e^{\alpha_{c}^{\prime }}}{e^{\alpha_{c}^{\prime }}+e^{\beta_{c}^{\prime }}+e^{\gamma_{c}^{\prime }}},$$ (4)

$$\beta_{c}=\frac{e^{\beta_{c}^{\prime }}}{e^{\alpha_{c}^{\prime }}+e^{\beta_{c}^{\prime }}+e^{\gamma_{c}^{\prime }}},$$ (5)

$$\gamma_{c}=\frac{e^{\gamma_{c}^{\prime }}}{e^{\alpha_{c}^{\prime }}+e^{\beta_{c}^{\prime }}+e^{\gamma_{c}^{\prime }}}.$$ (6)

Where $\alpha_{c}$ $\beta_{c}$ and $\gamma_{c}$ are the c-th elements of α , β , and γ , respectively.

The final output feature maps Y are computed *via* combining various kernels with their attention weights:

$$Y_{c}=\alpha_{c}∙V^{\prime }+\beta_{c}∙V^{\prime \prime }+\gamma_{c}∙V^{\prime \prime \prime }\;$$ (7)

where α, β , and γ should satisfy $\alpha_{c}+\beta_{c}+\gamma_{c}=1$ and $Y=\left[Y_{1},Y_{2},\ldots ,Y_{c}\right],Y_{c}\in R^{H\times W}$ .

#### 2.3.2 Pixel-level multimodal fusion deep networks for the protein subcellular localization

**FIGURE 4:**
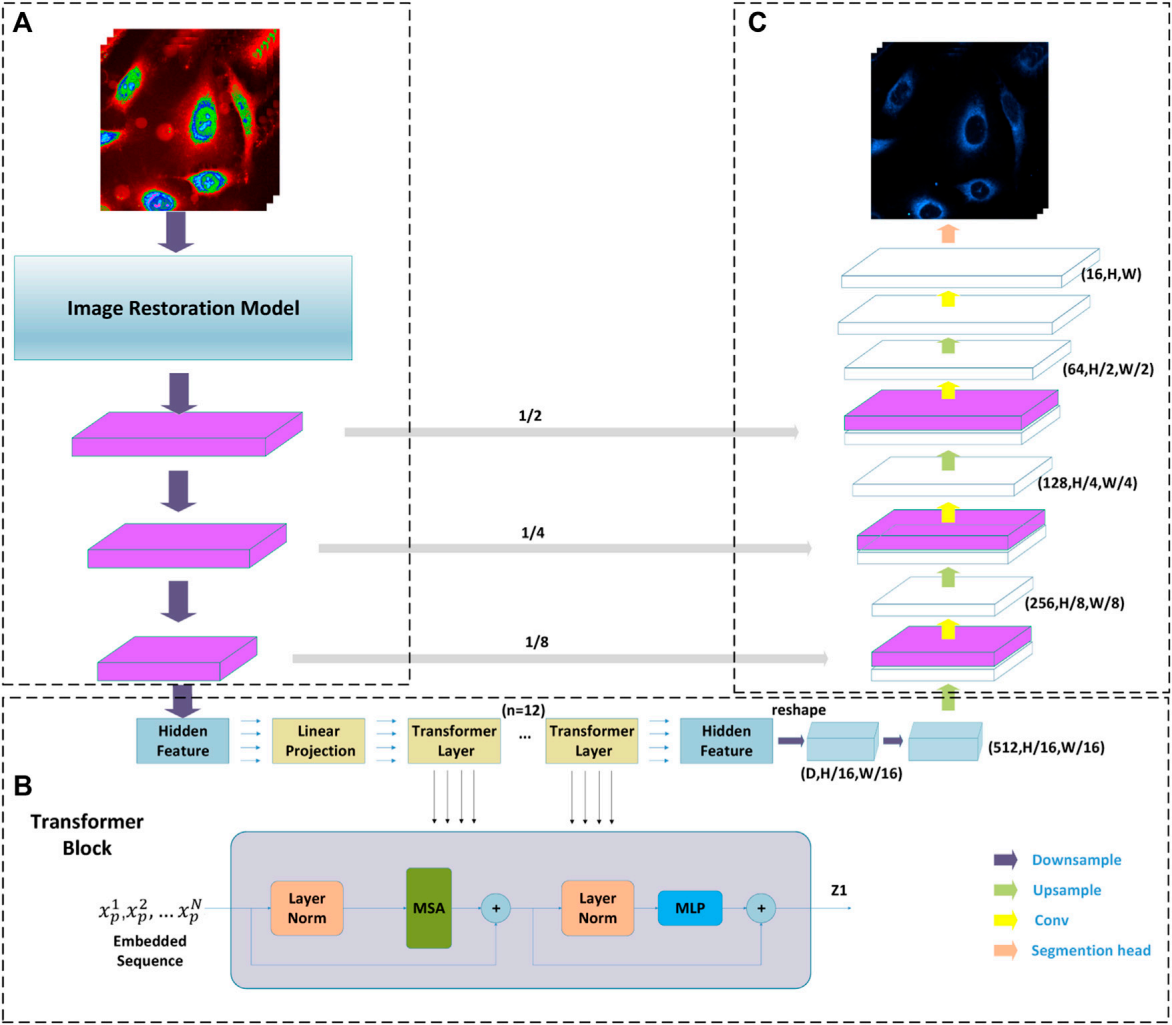
Diagram of the Transformation and CNN multimodel fusion-based network architecture underpinning presented tool. Part **(A)** is CNN blocks combined with an image restoration model; Part **(B)** is a transformer taking raw image as input; and part **(C)** is a classical Unet where the decoder takes the output of the transformer as its input.

As shown in Figure 4, the Transformer and Unet fusion model is constructed to predict the optical microscopy images by bridging CNN for extracting feature presentations and an efficient deformable Transformer for modeling the long-range dependency on the extracted feature maps. In our experiment, in the multi-layer perceptron (MLP) layers of the transformer model, the activation function GELU is replaced with ELU, which performs better because in medical images, negative values are as important as positive values, which is defined as (Witmer and Bhanu, 2022):

$$ELU=\left\{ \begin{array}{c} x,if\;x\geq 0\\ \propto e^{x}-1,if\;x<0, \end{array}\right.$$ (8)

where hyper parameter α is set to 1.

Given an H × W spatial resolution raw image with C channels, which are matrices as *X ∈ R* ^*H×W×C*^, the advantage of the Transformer and Unet fusion model-based image segmentation task lies in predicting the corresponding cell fluorescence images for different subcellular organelles. The input raw image X is first split into N non-overlapping 2D spatial patches with size P×P, which can be defined as { ${x_{p}^{i}\in R}^{P^{2}.C}$ *|i=1..., N*}, where $N=\frac{HW}{P^{2}}$ is the number of images. Then, the vectorized *x*_p_ is mapped to multi-dimensional feature spaces through a learnable linear patch-embedding projection ∅ . To maintain position information, the positions embedded ∅pos were added to patch features for encoding the spatial information of the patches, which is as shown (Wang et al., 2004; Manifold et al., 2019):

$$z_{0}=\left[x_{p}^{1}\emptyset ;x_{p}^{2}\emptyset ;\cdots \cdots ;x_{p}^{1N}\emptyset \right]+\emptyset pos,$$ (9)

where ${\varphi_{pos}\in R}^{N\times D}$ is the position embedding; $x_{p}^{1}$ *,* $x_{p}^{2}$ *,...* $x_{p}^{1N}$ represent the vectorized patches; and N is the size of non-overlapping patches. ${\emptyset \in R}^{\left(P^{2}.C\right)\times D}$ is the learnable patch embedding projection, C is the channel number of the input raw image X, and P is the size of the input raw image X.

In these cases, as shown in part B in Figure 2, the Transformer block containing K = 12 Transformer layers in the encoder part is used to extract the features. For the *k*th transformer layer, which is mainly composed of a multi-head self-attention module and a multi-layer perception module. The output of each layer can be defined as follows (Wang et al., 2004; Manifold et al., 2019).

$${\hat{v}}_{k}=MHSA\left(NM\left(v_{k-1}\right)\right)+v_{k-1},$$ (10)

$$v_{k}=MLP(NM({\hat{v}}_{k})+{\hat{v}}_{k},$$ (11)

where *NM*(·) is the layer normalization and *v*_k_ denotes the encoded image representation.

The MHSA (.) is defined as follows (Donovan-Maiye et al., 2018):

$$MHSA\left(\alpha ,\beta ,\mu \right)=Cat\left(h_{1},h_{2},\cdots \cdots {,h}_{Nh}\right)W^{O},$$ (12)

$$s.t.h_{i}=Atf\left(\alpha P_{i}^{\alpha },\beta P_{i}^{\beta },\mu P_{i}^{\mu }\right),$$ (13)

$$Atf\left(\alpha ,\beta ,\mu \right)=softmax\left(\frac{\alpha \beta^{T}}{\sqrt{n_{k}}}\right)\mu ,$$ (14)

where α is the query vector, *β* denotes the key vector, and μ denotes the value vector of the input maps. *P*_*O*_ is the projection matrix of the output vector, P_α_ denotes the projection matrix of the query vector, *P*_*β*_ denotes the projection matrix of the key vector, P_μ_ denotes the projection matrix of the value vector, and *n*_*k*_ denotes the dimensions of *α* and *β*.

### 2.4 Dataset

We employed a subset of SRS images in the fixed lung cancer cell (A549, from ATCC) data set as one of pre-trained sources of data. These data sets were acquired simultaneously using ScanImage by collecting the SRS signals from lock-in amplifiers and fluorescence signals from photomultiplier tubes (Zhang et al., 2019). For the fluorescence signals, all dyeing schemes were based on the standards, provided that three different color fluorescent dyes were used to label and track the nucleus, mitochondria, and endoplasmic reticulum, respectively. The optical cell images with 512 × 512 pixels were obtained at a dwell time of 4 μs.

Another trained source data we employed are the dataset cell images which were acquired using GE’s IN Cell Analyzer systems (Esakkirajan et al., 2019). These data sets were applied to test different deep-learning methods and evaluate their performance.

## 3 Results

### 3.1 Experimental settings

To compare the performance of different models, the setting of experimental parameters should be as consistent as possible. First, the development, training, prediction, and image processing of all models are calculated by using the Pytorch platforms, and the graphics card of the server adopts the GeForce RTX 3080. Second, during model training, the value of momentum is set at 0.9, the value of the batch size is set to 8, and the weight attenuation for the training neural network is set to 1 × 10^−4^. At the same time, the maximum number of epochs for the contrasting models is set at 200. In order to select the initial learning rate, a series of values are computed to test its training effect in the model. According to the experimental comparison, it was proved that 0.001 was the best choice to set as the initial learning rate.

**FIGURE 5:**
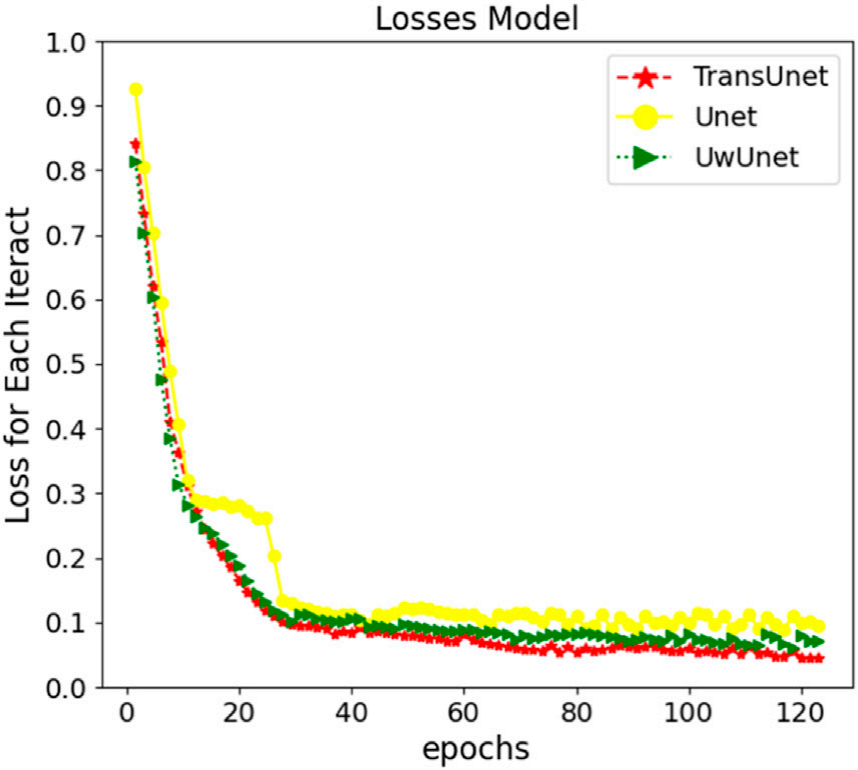
The neural network training curves for three prediction models which are Unet, UwUnet, and PLMF-net.

The neural network training curves for three different prediction methods are shown in Figure 5. For a better performing Transformer and Unet fusion model, as Figure 5 depicts, the training process only took about 120 steps until the training accuracy increased over 96%. The error decay in Figure 5 demonstrates that the method with the PLMF-net mechanism gained better performance on different training samples, in comparison with the classical Unet and UwUnet models, where our strategy avoids over-fitting because the error does not increase with the change of the training mode, and the error attenuation remains stable.

### 3.2 Metrics for performance evaluation

In order to verify the credibility of predictions, five quantified metrics are applied in measuring the performance of different prediction algorithms. All the evaluation metrics mentioned previously can be consecutively calculated as follows.

The accuracy (AY) and overall accuracy (OA) are common standard metrics for predicting subcellular locations, which can been calculated as follows:

$$AY\left(i\right)=\frac{R\left(i\right)}{S\left(i\right)},$$ (15)

$$OA=\frac{\sum_{i=1}^{10}R\left(i\right)}{\sum_{i=1}^{10}S\left(i\right)},$$ (16)

where *R*(*i*) is the correctly predicted values in the *i*th subcellular locations, and S(*i*) represents the total values in the *i*th subcellular locations.

Mean intersection over union (MIoU) is another standard metric for segmentation purposes (Rahman et al., 2022). Intersection over union (IoU) is a ratio computed on a per-class basis between the ground truth and the protein subcellular location prediction. Mean intersection over union (MIoU) is the average of the IoU ratio which can been calculated as follows:

$$IoU=\frac{T⋂P}{T⋃P},$$ (17)

$$MIoU=\frac{1}{k+1}\overset{k}{\underset{i=0}{\sum }}\frac{p_{ii}}{\sum_{j=0}^{k}p_{ij}+\sum_{j=0}^{k}p_{ji}-p_{ii}},$$ (18)

where it is assumed that the total number of classes is (*k* + 1), and *p*_ij_ is the amount of pixels of class *i* inferred to class *j*. *p*_ii_ represents the number of true positives, while *p*_ij_ and *p*_ji_ are usually interpreted as false positives and false negatives, respectively.

Pearson’s correlation coefficient (PCC) (*r*_py_ ∈ [−1, 1]) is another metric which give the relationships between the feature values and the predicted values by measuring the correlation between the pixels of the true and predicted images. Given N sample pairs {(*p*_1_, *y*_1_),..., (*p*_N_, *y*_N_)}, we can get:

$$r_{py}=\frac{\sum_{i=1}^{N}\left(p_{i}-\bar{p}\right)\left(y_{i}-\bar{y}\right)}{\sqrt{\left(\left(\sum_{i=1}^{N}{\left(p_{i}-\bar{p}\right)}^{2}\right)\left(\sum_{i=1}^{N}{\left(y_{i}-\bar{y}\right)}^{2}\right)\right)}}$$ (19)

where $\bar{p}$ and $\bar{y}$ are the sample means. Note that when *p*_i_ and *y*_i_ are binary, *r*_py_ becomes the Matthews correlation coefficient which is known to be more informative than the F_1_ score (Dice coefficient) on imbalanced datasets.

MSE (mean square error) is a function that is used to evaluate the difference between the targeted values and the predicted values (Voronin et al., 2020). RMSE (root mean square error) further evaluates the spatial detail information between images, while NRMSE (normalized root mean square error) normalizes RMSE for easier observation and comparison. For the image prediction work, the NRMS can be applied in computing the accuracy between the pixel in the predicted image and the same pixel in the truth image, which was obtained by:

$$MSE=\frac{1}{M\times N}\overset{M}{\underset{i=1}{\sum }}\overset{N}{\underset{j=1}{\sum }}{\left(u^{\prime }\left(i,j\right)-u\left(i,j\right)\right)}^{2},$$ (20)

$$RMSE=\sqrt{MSE\left(u^{\prime },u\right),}$$ (21)

$$NRMSE=\frac{\sqrt{\frac{1}{M\times N}\overset{M}{\underset{i=1}{\sum }}\overset{N}{\underset{j=1}{\sum }}{\left(u^{\prime }\left(i,j\right)-u\left(i,j\right)\right)}^{2}}}{{u^{\prime }\left(i,j\right)}_{\max}-{u^{\prime }\left(i,j\right)}_{\min},},$$ (22)

where $u^{\prime }\left(i,j\right),u\left(i,j\right)$ represent the image to be evaluated and the original image, respectively. N represents the length and width of the image.

The peak signal to noise ratio (PSNR) is the most commonly metric used in the image quality assessment, which can be obtained by:

$$PSNR=10\log_{10}\left(\frac{m_{x}\times m_{y}\times V_{\max}^{2}}{\sum_{r,t}{\left[t\left(x,y\right)-d\left(x,y\right)\right]}^{2}}\right),$$ (23)

where V_max_ denotes the maximum predicted value of the source image. *t(x,y)* is the matrix of the raw-source image, *d(x,y)* is the matrix of the noise-removed image, and (*x,y*) denotes the pixel coordinate in an given *m*_x_ × *m*_y_ image.

Structural similarity index (SSIM) can be used as a quality evaluation index for similarity comparison among image prediction results, which can be obtained by:

$$s\left(x,y\right)=\frac{\sigma_{xy}+c_{3}}{\sigma_{x}\sigma_{y}+c_{3}},$$ (24)

$$l\left(x,y\right)=\frac{{2\mu }_{x}\mu_{y}+c_{1}}{\mu_{x}^{2}+\mu_{y}^{2}+c_{1}},$$ (25)

$$c\left(x,y\right)=\frac{{2\sigma }_{x}\sigma_{y}+c_{2}}{\sigma_{x}^{2}+\sigma_{y}^{2}+c_{2}}.$$ (26)

The SSIM value is calculated for two signals as well as for images after combining Eqs 24 and 25 as:

$$SSIM\left(x,y\right)={\left[l\left(x,y\right)\right]}^{m}{\left[c\left(x,y\right)\right]}^{n}{\left[s\left(x,y\right)\right]}^{p}$$

$$=\frac{\left(2\mu_{x}\mu_{y}+C_{1}\right)\left(2\sigma_{xy}+C_{2}\right)}{\left(\mu_{x}^{2}+\mu_{y}^{2}+C_{1}\right)\left(\sigma_{x}^{2}+\sigma_{y}^{2}+C_{2}\right)}$$ (27)

where *m*, *n*, and *p* denote the magnitude values of the structure component *s(x,y)*, the luminance component *l(x,y),* and the contrast component *c(x,y),* respectively. *µ*_*x*_ *and µ*_*y*_ are the average of *x*_*i*_ *, y*_*i,*_ respectively. *σ*_*x*_ and *σ*_*y*_ are the variance of *x*_*i*_ and *y*_*i*_, respectively.

### 3.3 Comparison among the different methods for image denoising and restoration

**FIGURE 6:**
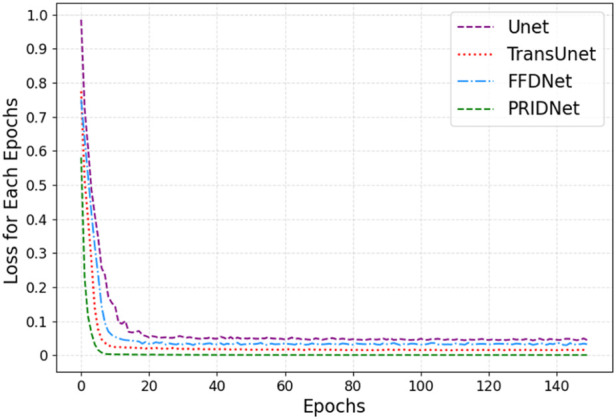
The neural network training curves for four denoising models which are Unet, TransUnent, FFDNet, and PLMF-net.

In this work, we mainly focus on applying various deep-learning methods to significantly enhance the quality of non-linear optical images. A series of cellular images acquired using GE’s IN Cell Analyzer systems were tested in this work. The neural network training curves for four different restoration methods which are Unet, TransUnet, Fast and Flexible Denoising Convolutional Neural Network (FFDNet), and Pyramid Real Image Denoising Network (PRIDNet) module are shown in Figure 6. For better performing Transformer and Unet fusion model, as Figure 1 depicts, the training process only took about 140 steps until the training accuracy increased over 96%. The error decay in Figure 6 demonstrates that the method with the PRIDNet mechanism had better performance on different training samples in comparison with the Unet, TransUnet, and FFDnet models, where our strategy avoids over-fitting because the error does not increase with the change of the training mode, and the error attenuation remains stable.

**FIGURE 7:**
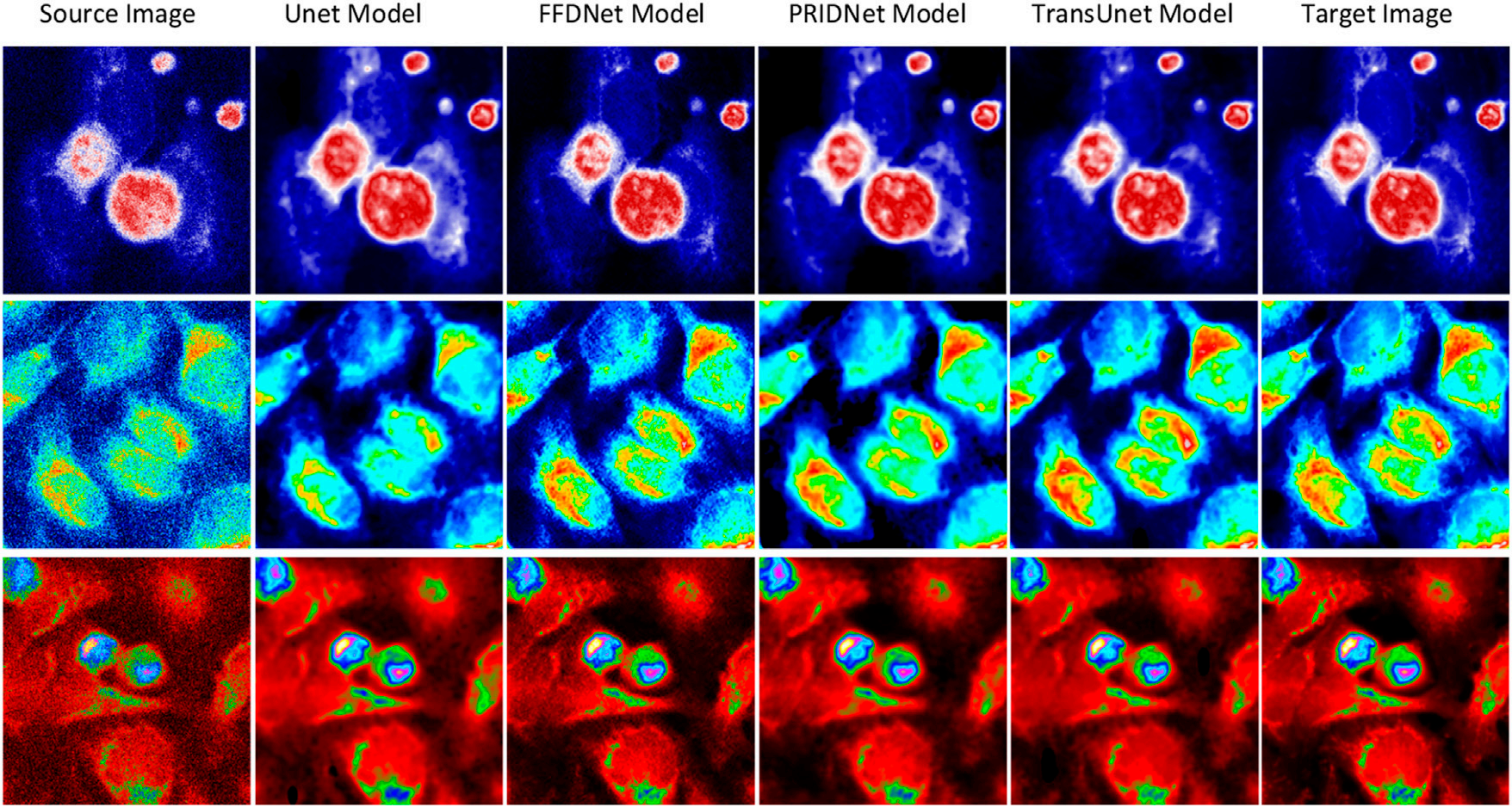
Results of deep-learning-based image denoising. Different stains and cell cultures. The first column shows the source optical images of Hela cells; the second column shows the ground truth optical images; and the following two columns display the denoising results by UNet and MultiFusion models, respectively.

For a better comparison, we built a set of raw-cell optical images to have a common ground truth, and the zero-mean Gaussian noise with independent identical distribution is mixed into the original image as the input feature map for training (seen in Figure 7). The performance comparison results of the various deep-learning-based denoise images related to subcellular detection with three different parameters (RMSE, PSNR, and SSIM) are also shown in Figure 7.

The denoising ability of different deep-learning algorithms based on Unet, TransUnet, FFDnet, and the denoising method based on PRIDNet is compared. This work further uses several metric indicators such as SSIM, RMSE, and PSNR values for quantitative analysis (seen in Table 1). As discussed previously, the Structural Similarity Index (SSIM) which is usually used as a representative image fidelity measurement by judging the structural similarity of two optical images based on three metrics, which are luminance, contrast, and structure, is a valuable and meaningful reference-based index for natural images. Another quantified metric PSNR used in this work to analyze the denoising effect for these different deep-learning methods. The higher the PSNR value, the higher the image fidelity. The quantified metric RMSE is also used to measure the accuracy of different deep-learning-based imaging-restoration methods relative to the truth data. From Table 1, it can be clearly found that the PRIDNet method is better than the Unet, TransUnet, and FFDnet denoising methods at different condition sets. The increase of PSNR from 28.398 to 31.716 dB, and the decrease of RMSE from 5.762 to 2.707 validate the remarkable resolution enhancement; meanwhile, the higher level SSIM index of 84.5% of the PRIDNet method compared to 77.5% of the Unet method proves the authenticity of reconstruction. Apparently, SSIM and PSNR of the PRIDNet network reconstruction are both better than that of the deconvolution results of Unet, TransUnet, and FFDnet-based denoising measurements. As discussed previously, it is proved that the PRIDNet method can help in cell-imaging restoration work and improve its denoising performance.

**TABLE 1:** The performance comparison results of the various deep learning-based denoised images related to subcellular detection with three different metrics (SSIM, RMSE, and PSNR).

Model	SSIM↑	RMSE↓	PSNR (dB)↑
Unet	0.775	5.762	28.398
FFDNet	0.817	4.731	30.683
TransUnet	0.842	3.240	31.469
PRIDNet	0.845	2.707	31.716

### 3.4 Comparison of the performance with various prediction models

In this section, it is investigation and comparison among different deep-learning models are conducted for predicting the subcellular organelle localization from label-free optical microscopy images. Even though the traditional imaging-based pipeline has cells stained, the SRS imaging can give more information on cell shape and subcellular structure without using molecular probes. At the same time, it also produces low-contrast and complex images, which makes it difficult to clearly indicate the biochemical features of these cells. So there exist some challenges of using these deep-learning-based methods to identify, segment, and quantify each subcellular structure in the cell’s optical image. As a result, some advanced analysis methods are needed to be developed for exploring the rich information hidden in a cell image. Based on the aforementioned reasons, the new PLMF-net method is proposed in this work which bridges the Transform model and convolutional neural networks to automatically segment organelles.

**FIGURE 8:**
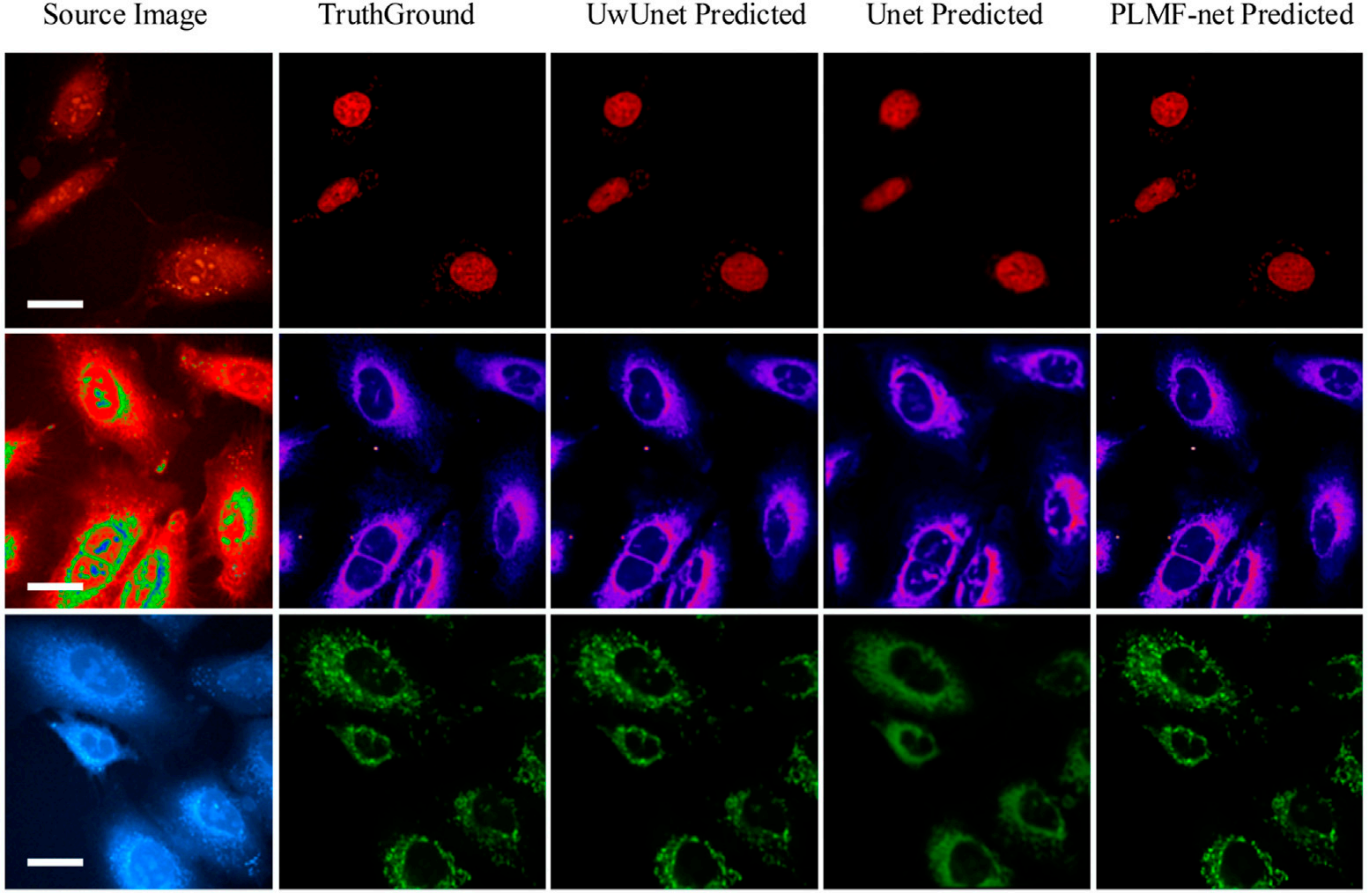
Predicted organelle fluorescence from hyperspectral SRS microscopy images by using different methods. The first column shows the input SRS image, the second column shows the ground-truth fluorescence image, and the following three columns display the predicted fluorescence results by UwUNet, U-Net, and PLMF-Net, respectively, for nuclei (top row), mitochondria (middle row), and endoplasmic reticulum (bottom row).

To demonstrate the application of the deep-learning model in label-free organelle prediction, we used fluorescence imaging of the fixed lung cancer cells as a ground-truth model and SRS microscopy images as the source-image model. As shown in Figure 8, the first column shows live cell Raman optical image, the second column is ground-truth fluorescence images taken after the cells that are stained, and the following three columns are predicted fluorescence cell images with the UwUnet method, Unet method, and PLMF-net method, respectively. From the experimental analysis results, we can see that the PLMF-net method can accurately predict the location of each organelle from cell-optical imaging data at the same time.

**FIGURE 9:**
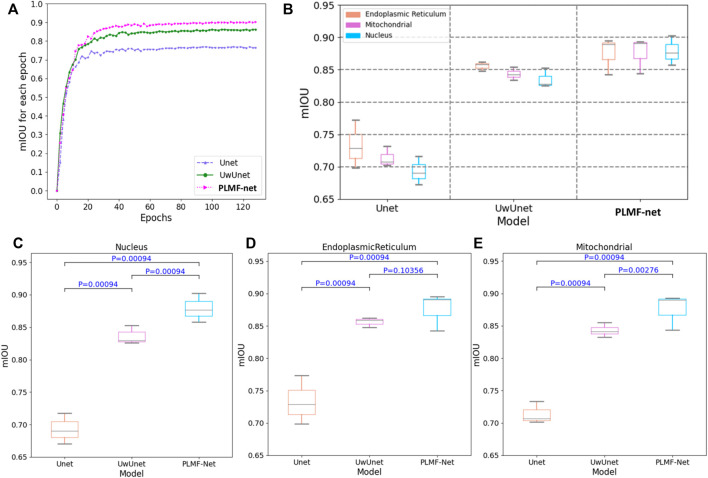
The mean intersection over union (mIoU) of different deep-learning models over all SRS microscopy images in the fixed lung cancer cell (A549, from ATCC) detection dataset. **(A)** mIoU for each epoch comparison among different methods. Unet (purple triangle dash line), UwUnet (green circle solid line), PLMFNet (pink arrow dotted line) represent Unet, UwUnet, and PLMFNet, respectively; **(B)** Box plot of mIoU accuracy over all organelle (nuclei, mitochondria, and endoplasmic reticuli) prediction task sets with the PLMFNet-based learning model and compared with that of various deep neural network‐based prediction models such as Unet and UwUnet learning models. **(C)** Box plot of mIoU accuracy on the nuclei prediction task set with PLMFNet-based learning model and compared with that of varied deep neural network‐based prediction models such as Unet and UwUnet learning models on all. Horizontal bars depict Mann–Whitney U tests for significance of differences in the mIoU value between different deep-learning models (corrected *p* < 0.001; not significant *p* > 0.05). **(D)** The box plot of mIoU accuracy on the endoplasmic reticuli prediction task set with the PLMFNet-based learning model and compared with that of varied deep neural network‐based prediction models such as Unet and UwUnet learning models. Horizontal bars depict the Mann–Whitney U tests for significance of differences in the mIoU value between different deep-learning models (corrected *p* < 0.001; not significant *p* > 0.05). **(E)** The box plot of mIoU accuracy on the mitochondria prediction task set with PLMFNet-based learning model and compared with that of varied deep neural network‐based prediction models such as Unet and UwUnet learning models. Horizontal bars depict Mann–Whitney U tests for significance of differences in the mIoU value between different deep-learning models (corrected *p* < 0.001; not significant *p* > 0.05).

In order to quantitatively compare and analyze the effects of different prediction methods, we calculated the several quantitative metrics to explore the differences between the predicted results and expected results of different methods, so as to compare the prediction performance between the methods proposed in this work with other classical methods. We first measured the accuracy of label-free prediction algorithms using the mean intersection over union (IOU) evaluation metric. Here, we used the box-plot graph to give a more visual and intuitive representation for the quantitative evaluation of mIOU parameters on the prediction results of different algorithms (Figure 9). The boxplot in Figure 9 shows five statistics in the data: minimum, first quartile, median, third quartile, and maximum. In the Figure 9, the minimum value is represented by the extension of the black lines at the bottom, while the maximum value is represented by the extension of the black line on the top. The range of these two black lines refers to the mIOU accuracy range. The top and bottom of the box refer to the accuracy of the upper quartile (=0.75) and lower quartile (=0.25), respectively. The gray solid line in the box indicates the median accuracy. It can be seen from Figures 9B–E that compared with other methods, the PLMFNet method achieves the best performance among all the nuclei, mitochondria, and endoplasmic reticuli datasets. Compared to the observed datasets, PLMFNet significantly performed favorably in metric mIoU with 0.902, 0.894, and 0.893 for the nuclei, mitochondria, and endoplasmic reticuli task sets, respectively, against alternative UwUnet approaches with mIoU 0.8520.861, 0.854. Specifically, the classical Unet approach performed significantly worse with mIoU 0.716, 0.771, 0.731, respectively.

**FIGURE 10:**
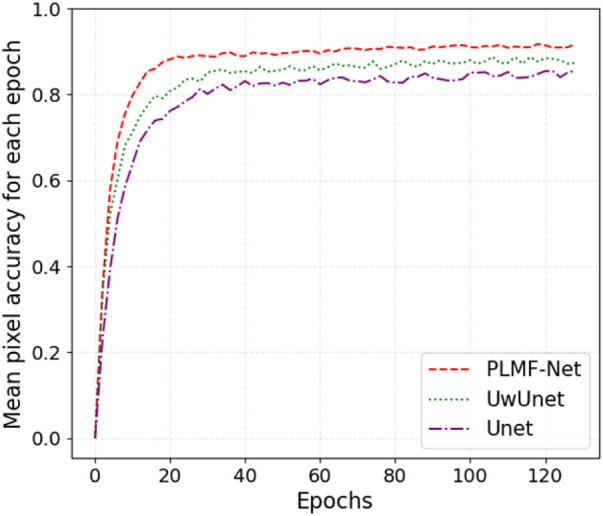
Mean pixel-accuracy comparison among different methods. The red dashed line, green dotted line, and purple dashed dotted lines represent the Unet, UwUnet, and PLMFNet models, respectively.

In addition, we also give a comparison of the prediction performance with the mean pixel accuracy curves among the Unet, UwUnet, and PLMFNet models as shown in Figure 10. One can observe from Figures 9, 10 that the PLMFNet ensemble method can achieve the highest mean pixel accuracy of 0.92.

To further characterize the predictive performance of the three variants of deep-learning-based predictor on the organelle (nuclei, mitochondria, and endoplasmic reticuli) segmentation task and to give comparable measures, we also provide cosine correlation performance metric to quantify the accuracy of the predictions. A cosine-similarity value is usually used to determine the degree of similarity between two non-zero vectors by measuring the cosine of the angle between them in the inner product space.

**FIGURE 11:**
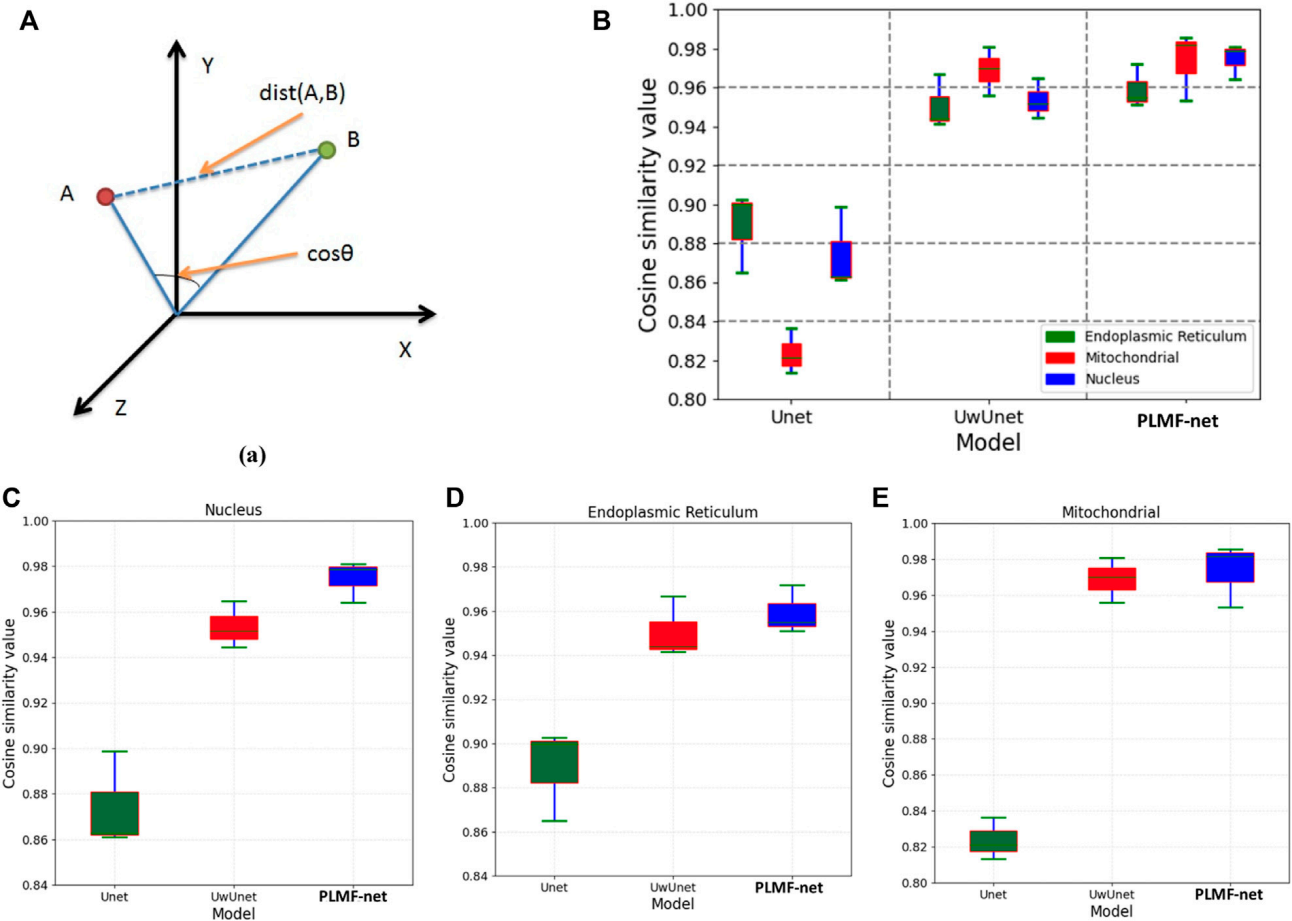
Comparison of the cosine correlation performance among various prediction algorithms. **(A)** The cosine correlation performance measures that are typical for predictive tasks. **(B)** The box plot of cosine similarity value over all organelle (nuclei, mitochondria, and endoplasmic reticuli) prediction task sets with the PLMFNet-based learning model and compared with that of varied deep neural network‐based prediction models such as Unet and UwUnet learning models. **(C)** The box plot of cosine similarity value on the nuclei prediction task set with the PLMFNet-based learning model and compared with that of varied deep neural network‐based prediction models such as Unet and UwUnet learning models. **(D)** The box plot of cosine similarity value on endoplasmic reticuli prediction task set with PLMFNet-based learning model and compared with that of varied deep neural network‐based prediction models such as Unet and UwUnet learning models. **(E)** The box plot of cosine similarity value on the mitochondria prediction task set with the PLMFNet-based learning model and compared with that of varied deep neural network‐based prediction models such as Unet and UwUnet learning models.

Compared with Pearson similarity measure, the adjusted cosine similarity metric is an improved evaluation index and a modified form of vector-based similarity. It makes up for the disadvantage that different users may have different scoring schemes just like some users may generally give a higher evaluation of the project, while others may give a lower evaluation of the project. In order to eliminate the disadvantage of vector-based similarity, the adjusted cosine-similarity measure subtracts the average score from the score of each user on related items. In principle, Pearson’s method will perform worse than the cosine similarity approach in contexts where users tend to have very differing sets of items in their profiles. It is because that the cosine similarity approach provides a kind of Bayesian regularization for the metric, ensuring that the similarity is not completely determined by the item subset (which may be very small) jointly owned by two users, while this does not exist in Pearson correlation although it can be added by significance weighting Figure 11A.

Compared with other methods, it can be seen in Figures 11B–E that the PLMFNet method achieved the top performance on all the nuclei, mitochondria, and endoplasmic reticuli datasets. Compared to the observed datasets, PLMFNet significantly performed favorably in the metric cosine similarity value with 0.978, 0.982, 0.957 for the nuclei, mitochondria, and endoplasmic reticuli task sets, respectively, against alternative UwUnet approaches with cosine similarity values of 0.951, 0.969, and 0.943. Especially, the classical Unet approach performed significantly worse with cosine similarity values of 0.862, 0.821, and 0.883, respectively.

Moreover, in terms of giving an explicit and quantitative analysis, the details of the evaluation results are calculated in this work shown in the Tables 2–4. For different prediction models, three different quantitative parameters are computed to compare and analyze the accuracy of protein subcellular localization from label-free live-cell imaging. As can be seen from Tables 2–4, it presents the label-free prediction results of three variants of deep-learning-based predictor on organelle (nuclei, mitochondria, and endoplasmic reticuli) segmentation task, in terms of quality metric values with NRMSE, PCC, and mean IoU. Comparing with the Unet method and UwUnet prediction methods, our proposed method PLMFNet surpasses the Unet and UwUnet methods on all quality metric values with NRMSE, PCC, and mean IoU. Especially for the nuclei prediction task, PLMFNet achieves a 5.0% improvement over the UwUnet method and 18.6% of mIoU over the Unet method in terms of mIoU, and for the mitochondria prediction task, PLMFNet achieves a 3.9% improvement over the UwUnet method and 16.2% of mIoU over the Unet method in terms of mIoU. To sum up, through the comprehensive analysis of mIOU quantitative indicators corresponding to different methods in Tables 2–4, we can draw a more accurate conclusion from the quantitative standard that our method is the best of all methods.

**TABLE 2:** Comparison of quality measures for labeling-free prediction results with the PLMFnet model.

Organelle	Our method model		
	NRMSE↓	PCC↑	mIOU↑
Nucleus	0.193 ± 0.007	0.920 ± 0.002	0.902 ± 0.006
Endoplasmic reticulum	0.206 ± 0.009	0.924 ± 0.003	0.894 ± 0.005
Mitochondria	0.214 ± 0.002	0.911 ± 0.005	0.893 ± 0.004

Here, ↓ indicates that the lower the index value, the better the performance.↑ indicates that the higher the index value, the better the performance of the model.

**TABLE 3:** The prediction result measure of protein subcellular localization using the UwUnet model.

Organelle model	UwUnet method		
	NRMSE↓	PCC↑	mIOU↑
Nucleus	0.201 ± 0.002	0.892 ± 0.002	0.852 ± 0.008
Endoplasmic reticulum	0.225 ± 0.003	0.903 ± 0.005	0.861 ± 0.003
Mitochondria	0.217 ± 0.004	0.880 ± 0.006	0.854 ± 0.005

Here, ↓ indicates that the lower the index value, the better the performance.↑ indicates that the higher the index value, the better the performance of the model.

**TABLE 4:** Comparison of quality measures for labeling-free prediction results with the Unet model.

Organelle model	Unet model		
	NRMSE↓	PCC↑	mIOU↑
Nucleus	0.442 ± 0.003	0.843 ± 0.004	0.716 ± 0.006
Endoplasmic reticulum	0.454 ± 0.002	0.856 ± 0.009	0.771 ± 0.008
Mitochondria	0.511 ± 0.007	0.835 ± 0.005	0.731 ± 0.004

Here, ↓ indicates that the lower the index value, the better the performance.↑ indicates that the higher the index value, the better the performance of the model.

Furthermore, not only is the mIOU metric used as the evaluation index, more quantitative indicators are also utilized to compare and analyze the performance of different prediction models in this section. Table 2 shows that on the nuclei prediction test set, the obtained NMSE of PLMFNet model is 0.193, which has less than half that of the classical UNet model, and 3.98% improvement compared to UwUnet model. On the mitochondria-prediction test set, one can observe that the lowest NMSE value is acquired by the PLMFNet model as 0.217, which achieves a 55% improvement compared to the classical UNet model, and an 8.44% improvement compared to the UwUnet model. As for the mitochondria prediction test set, the obtained NMSE of the PLMFNet model is also lowest at 0.214 which has less than half that of the classical UNet model and 1.38% improvement compared to UwUnet model.

In order to further explore the prediction performance of different models, we give more calculations to correlate the pixels for the obtained organelle fluorescence images and the predicted organelle fluorescence from SRS microscopy images with three variants of the deep learning-based predictor, respectively. Another quantitative parameter PCC is also applied in detecting the consistency between the prediction results and the target values, so as to further study the variability. From Tables 2–4, it can be observed that the PLMFNet model shows the top performance in terms of the PCC coefficient. The predicted PCC value of the nuclear validation set is as high as 0.92 for our proposed PLMFNet model. Similar results were also observed in mitochondrial samples and the endoplasmic reticuli test set (Pearson’s *r* = 0.911 and 0.924, respectively). In terms of the nuclei, mitochondria, and endoplasmic reticuli test sets, the PCC similarity coefficient results from PLMFNet are all higher than the classical UNet performance as follows: 6.99%, 6.95%, and 4.44%, and the PCC similarity coefficient results from PLMFNet are all higher than the UwUNet performance as follows: 3.14%, 3.52% and 2.33%.

## 4 Discussions

For each case prediction of SRS microscopy images in the fixed lung cancer cell-detection dataset, differences of the median IOU between cases and deep-learning models were calculated, and statistical significance was determined using with the Mann–Whitney U test (Figure 9). As shown in Figures 9C–E, the Mann–Whitney U test was used to determine significant differences between each assessment of mIOU quantitative indicators against the same dataset in another prediction method. Compared with the Unet model, the PLMFNet model showed significant statistical difference in the mIOU value for predicting the nucleus, endoplasmic reticulum, and mitochondria (All *p* < 0.001). Compared with the UwUnet model, the PLMFNet model showed significant statistical difference in the mIOU value for predicting the nucleus and mitochondria (*p* < 0.01), but it depicted no statistical difference in the mIOU value for predicting the endoplasmic reticulum (*p* > 0.05). For all analyses, the PLMFNet model displayed higher prediction levels in mIOU quantitative indicators of the nucleus, endoplasmic reticulum, and mitochondria validation sets to the Unet and UwUnet methods on mean IoU metric values (Figure 9).

Overall, we demonstrated that the PLMFNet-based predictor from label-free microscopy offers a powerful experimental platform for conducting protein subcellular localization of living-cell imaging. The experiment of this work mainly investigates the results of the labeling-free method based on deep learning for protein subcellular localization from femtosecond-stimulated Raman spectroscopic microscope images. Compared with other classical optical-imaging methods, the stimulated Raman spectroscopy imaging has the advantages of not requiring fluorescent molecular markers and obtaining more information. However, this rich and overlapped information in the same collected image also brings difficulties of image analysis and feature extraction. Though a few of the label-free staining methods based on Raman imaging have been proposed and show promising results in some organelles, there is still a lack of rich and effective means to predict the subtle changes of the Raman spectra for single organelles.

**FIGURE 12:**
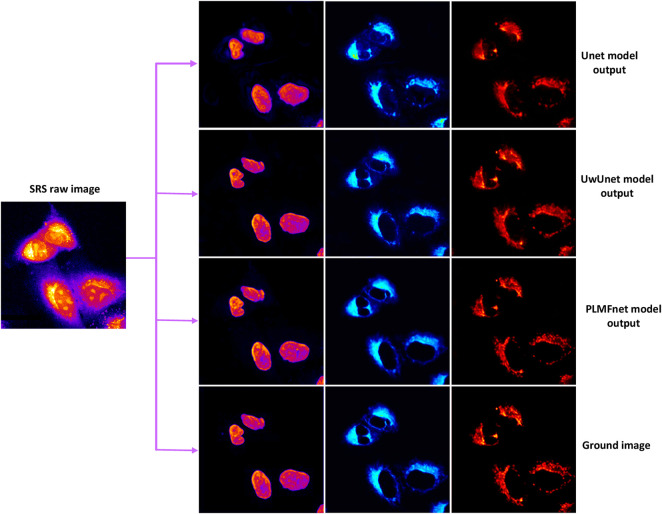
Subcellular localization prediction results of differentiation model from the label-free cell image experiments. The results for different organelles’ locations include nuclei (second column), mitochondria (third column), and endoplasmic reticulum (right) from the single raw SRS-imaging cell (left).

The results for subcellular localizations can be seen in Figure 12. One SRS raw image (left) for lung cancer (A549, from ATCC) cells was output from different deep-learning models at the same time to determine the accuracy of subcellular localization predictions which include nuclei (second column), mitochondria (third column), and endoplasmic reticulum (right). To sum up, through the comprehensive analysis of all three quantitative indicators in Figure 12 and Tables 2–4, we can draw a more accurate conclusion from the quantitative standard that our method is the best among all modules in Tables 2–4. In conclusion, our results show that deep learning creates some new opportunities for accurately predicting the location of cellular organelles from label-free cell optical images. Compared with the existing U-net-based medical image-prediction methods that are insufficient in catching on long-range dependencies in tested images, the pixel-level multimodal fusion predictor combines the merits of the Transform and UNet methods. The new multimodal fusion method can intelligently reveal and extract the non-linear correlation between features, so as to improve the performance of prediction. Additionally, as illustrated in Section 3.3, our deep-learning approach also improves the image SNR, which in addition offers a solution to highly suppress image artifacts and solve the distortion problems for high-speed SRS cell imaging.

## 5 Conclusion

In this work, we introduced a pixel-level multimodal fusion deep-network methodology which organically fuses the CNN branch and Transformer branch for efficiently predicting the location of cellular organelles from label-free cell optical images. The performance of the proposed pixel-level multimodal method was estimated and compared with other deep-learning models such as UwU net and Unet methods. It is shown from the experimental results that the new pixel-level multimodal fusion deep networks have top prediction performance, suggesting that they have great potential in the subcellular prediction of label-free cell optical images. All these experimental results proved that compared with previous CNN-based self-attention segmentation methods which lacked understanding of long-range dependencies in the image, the proposed predictor can encode strong global context by extracting the image features as sequences and utilize the low-level CNN features with a U-shaped hybrid architectural scheme that helps in improving the prediction accuracy. While our study focused on stimulated Raman scattering (SRS) microscopy, one could apply the same analytic procedure to other label-free optical-imaging instruments such as the bright field, phase, and differential interference contrast (DIC). In future work, we will further develop more advanced deep-learning methods to the hybrid Transform and Unet method, so as to further improve the performance of the protein subcellular location on cell optical imaging.

## Data Availability

The original contributions presented in the study are included in the article/Supplementary Material; further inquiries can be directed to the corresponding author.

## Fundings

The work is supported by the fund of the Beijing Municipal Education Commission, China, under grant number 22019821001, and the fund of Climbing Program Foundation from Beijing Institute of Petrochemical Technology (Project No. BIPTAAI-2021007).
